# Effects of GLP‐1 Receptor Agonists on Muscle Mass, Strength, and Quality in MASLD: A Systematic Review

**DOI:** 10.1111/liv.70643

**Published:** 2026-04-17

**Authors:** Fernando Iorra, Tanya Jayakar, Michael Yee, Mark R. Thursz, Beatriz D. Schaan, Pinelopi Manousou

**Affiliations:** ^1^ Postgraduate Program in Medical Sciences: Endocrinology Universidade Federal Do Rio Grande Do Sul Porto Alegre Brazil; ^2^ Faculty of Medicine, Imperial College London London UK; ^3^ Section of Endocrinology and Metabolic Medicine, St Mary's Hospital Imperial College NHS Trust London UK; ^4^ Liver Unit/Division of Digestive Diseases, Department of Metabolism, Digestion and Reproduction, Faculty of Medicine Imperial College London London UK; ^5^ Hospital De Clínicas De Porto Alegre Porto Alegre Brazil

**Keywords:** glucagon‐like Peptide‐1 receptor agonists, muscle strength, muscles, non‐alcoholic fatty liver disease

## Abstract

**Background and Aims:**

GLP‐1 receptor agonists (GLP‐1RAs) promote significant weight loss, but their impact on muscle mass, strength, and quality in metabolic dysfunction–associated steatotic liver disease (MASLD), a condition prone to muscle impairment, remains uncertain.

**Methods:**

We conducted a systematic review of MEDLINE/PubMed, Embase, and the Cochrane Library up to 2 December 2025. Interventional and observational studies assessing GLP‐1RA therapy and muscle‐related outcomes in adults with MASLD were included. Muscle mass was assessed by computed tomography or magnetic resonance imaging, dual‐energy X‐ray absorptiometry, or bioelectrical impedance analysis; lean mass and fat‐free mass were considered proxies. Muscle strength was evaluated using handgrip strength (HGS) or sit‐to‐stand (STS) tests. Muscle quality was assessed by imaging‐based quantification of intramuscular fat infiltration (myosteatosis) or functionally based on performance relative to muscle mass. Results were synthesised narratively.

**Results:**

Twelve studies were included (*n* = 810). Eleven assessed muscle mass (*n* = 785) across different GLP‐1RA regimens and treatment durations (12–52 weeks). Findings suggested relative preservation of muscle mass, with modest reductions generally proportional to overall weight loss. Muscle strength was evaluated in three studies (*n* = 477), with no evidence of deterioration in HGS or STS performance despite meaningful weight loss. Muscle quality was assessed in four studies (*n* = 139) using imaging or functional metrics, with results suggesting preserved or improved muscle quality. Evidence was limited by small sample sizes and heterogeneous assessment methods.

**Conclusion:**

Current evidence suggests that GLP‐1RA therapy is not associated with clinically meaningful loss of muscle mass or strength in adults with MASLD, with early data indicating possible improvements in muscle quality.

## Key Points

- Metabolic dysfunction–associated steatotic liver disease (MASLD) is associated with sarcopenia and impaired muscle function.
- GLP‐1 receptor agonists (GLP‐1RAs) induce significant weight loss, raising concerns about potential muscle loss.
- Across available studies, GLP‐1RA therapy was not associated with a clinically meaningful decline in muscle mass or strength.
- Early evidence suggests possible improvements in muscle quality through reductions in myosteatosis.
- Resistance exercise and adequate protein intake remain important to preserve muscle function.

## 1 Introduction

The bidirectional relationship between metabolic dysfunction‐associated steatotic liver disease (MASLD) and muscle health, encompassing muscle mass, strength, and quality, is increasingly recognised as the “liver‐muscle axis” [1]. Hepatic dysfunction has been demonstrated to exacerbate muscle catabolism, while impaired muscular conditioning can compromise metabolic health and potentially worsen liver disease [2, 3]. Accordingly, individuals with MASLD frequently exhibit markers of muscle impairment, including a higher prevalence of sarcopenia [4] and a reduced grip strength‐to‐BMI ratio [5]. This interplay is particularly relevant, as compromised muscle health in MASLD has been linked to adverse clinical outcomes [3, 6].

Central to the shared pathophysiology of MASLD and muscular decline are diabetes and obesity‐related drivers, such as insulin resistance, chronic low‐grade inflammation, and adipose tissue dysfunction [1]. These factors not only promote hepatic steatosis but also impair muscle protein synthesis and quality, often culminating in sarcopenic obesity [7, 8, 9]. Lifestyle factors, including physical inactivity, may further compromise both liver and muscle integrity [2, 10, 11]. Nevertheless, MASLD itself appears to play a direct role in muscle impairment, independently of obesity and insulin resistance [12]. From a clinical perspective, preservation of skeletal muscle remains crucial, as it represents the main insulin‐dependent site for glucose disposal [13], playing a central role in maintaining metabolic homeostasis and attenuating long‐term metabolic risk in this population.

One of the most clinically relevant manifestations of this muscle impairment is sarcopenia. This condition is characterised as a skeletal muscle disorder primarily defined by impaired muscle strength, which is considered the most reliable indicator of muscle function and the strongest predictor of adverse outcomes. The diagnostic algorithm proposed by the European Working Group on Sarcopenia in Older People (EWGSOP2) includes confirmation of low muscle quantity or quality as a second step after identifying reduced muscle strength [14]. However, assessing these parameters remains challenging, particularly muscle quality, which lacks a universally accepted definition and standardised measurement methods. Muscle quality encompasses structural and compositional features that influence muscle function—such as intramuscular lipid infiltration—as well as functional aspects, which can be conceptualised as muscle performance relative to muscle mass [14].

Recent advancements in the therapeutic management of obesity, particularly glucagon‐like peptide‐1 receptor agonists (GLP‐1RAs), have shown significant potential in mitigating MASLD by targeting its metabolic drivers [15, 16]. While GLP‐1RAs primarily act through glucose‐dependent insulin secretion, appetite regulation, and improvements in systemic insulin sensitivity, their direct effects on skeletal muscle remain an area of investigation. Preclinical evidence suggests that GLP‐1RAs may favourably impact muscle integrity by modulating myogenic factors, attenuating protein catabolism, and reducing local inflammation [17]. At the same time, concerns persist regarding the loss of muscle mass commonly observed during substantial weight reduction [18, 19]. Given the potential vulnerability of individuals with MASLD to muscle wasting and the widespread use of GLP‐1RAs in this population, clarifying how these agents influence muscle‐related outcomes is clinically relevant. This systematic review aims to evaluate how GLP‐1RA therapy affects muscle mass, strength, and quality in adults with MASLD.

## 2 Methods

### 2.1 Eligibility Criteria

We included both randomised and non‐randomised studies, conducted in individuals with MASLD (or previous designations of the disease, e.g., NAFLD/MAFLD). To be considered eligible, the study should have evaluated an intervention with a GLP‐1RA (such as semaglutide, liraglutide, dulaglutide, or exenatide) or a GLP‐1/GIP Dual Agonist (such as tirzepatide). Additionally, it should have provided an assessment of any muscle outcome,e.g., muscle quantity/mass or muscle strength. As a secondary outcome, we also considered studies reporting muscle quality data, if available.

Studies reporting lean mass (e.g., total lean mass, appendicular lean mass) were included as proxies for muscle quantity, given their widespread use in clinical settings and body composition assessments. Tools for evaluating outcomes included, but were not limited to, body composition or muscle mass assessments based on magnetic resonance imaging (MRI), computed tomography (CT), dual‐energy X‐ray absorptiometry (DXA), or bioelectrical impedance analysis (BIA); and muscle strength assessments based on handgrip strength (HGS) or sit‐to‐stand (STS) tests. In evaluating studies on muscle quality, different imaging‐based techniques were considered. These included methods derived from magnetic resonance imaging (MRI), such as proton density fat fraction (MRI‐PDFF) obtained via multi‐echo Dixon sequences or related techniques, which directly quantify muscle fat fraction, and proton magnetic resonance spectroscopy ([1] H‐MRS), which allows for the quantification of both intramyocellular and extramyocellular lipid content in vivo [20]. Alternatively, muscle quality was evaluated using functional metrics, such as the muscle strength‐to‐mass ratio.

Studies conducted in animals or in vitro were excluded, as well as those involving individuals under 18 years or pregnant women. Case reports, reviews, and editorials were not considered. No language restrictions were established. We did not include conference abstracts.

### 2.2 Information Sources, Search Strategy and Study Selection

We searched MEDLINE/PubMed, Embase, and the Cochrane Library, from inception to 2 December 2025, according to a structured search strategy specific to each database (Table S1). The libraries were merged in Covidence (Covidence systematic review software, Veritas Health Innovation, Melbourne, Australia), after which duplicates were removed. A pair of reviewers (FI and TJ) independently evaluated the eligibility of titles and abstracts, remaining blinded to each other's decisions during this stage. Conflicts were resolved by consensus or by a third investigator (PM). Full‐text evaluations followed the same independent and blinded protocol.

### 2.3 Data Collection Process

Data extraction was performed independently and in duplicate (FI and TJ) using a Microsoft Excel spreadsheet, with reviewers blinded to each other's extraction until the final comparison. Extracted information included publication date, title, authors, year of publication, study design, participant characteristics (mean age, sex, comorbidities), sample size, type and dosage regimen of GLP‐1RA, comparator or control group, weight and body mass index (BMI), quantification of muscle mass, strength or quality, and the specific assessment tool used for each muscle‐related outcome.

For studies reporting multiple follow‐up periods, data from the longest available follow‐up were extracted. In crossover randomised trials, only pre‐crossover data were included in the analysis. When relevant data were missing, study authors were contacted to request the original numerical information.

### 2.4 Study Risk of Bias Assessment

The risk of bias assessment was performed independently by two reviewers (FI and TJ). Disagreements were resolved through consensus or, when necessary, by consultation with a third reviewer (PM). The Risk of Bias 2 (RoB 2) tool was applied to randomised controlled trials (RCTs), while the Risk of Bias in Non‐randomised Studies of Interventions (ROBINS‐I) tool was used for non‐randomised studies of intervention (NRSIs). These instruments provide a structured and comprehensive evaluation of potential sources of bias across key methodological domains, including randomisation processes, deviations from intended interventions, missing data, outcome measurement, and selective reporting.

### 2.5 Data Synthesis and Statistical Analysis

This systematic review was reported in accordance with the Preferred Reporting Items for Systematic Reviews and Meta‐Analyses (PRISMA) [21]. Given the heterogeneity of the included studies and the impossibility of performing meta‐analysis, a structured qualitative synthesis was conducted following the principles of the Synthesis Without Meta‐analysis (SWiM) extension [22]. Standard errors were converted to standard deviations whenever required to harmonise reporting across studies. The protocol was registered on PROSPERO (CRD420251268768).

Results were summarised narratively and tabulated according to outcome domain—(i) muscle mass, (ii) muscle strength, and (iii) muscle quality—and further organised by assessment method within each domain. The narrative synthesis included the description of the direction of change in each outcome (reduction, no effect, or increase), as reported by the individual studies. For muscle mass, lean mass measures were included as proxies when reported; however, their physiological and technical differences from skeletal muscle mass were explicitly considered in the interpretation of findings. For studies reporting both total body weight and lean mass in baseline and follow‐up, we performed group‐level calculations to estimate the percentage of weight loss attributable to lean mass.

## 3 Results

### 3.1 Study Selection and Characteristics of Included Studies

**FIGURE 1:**
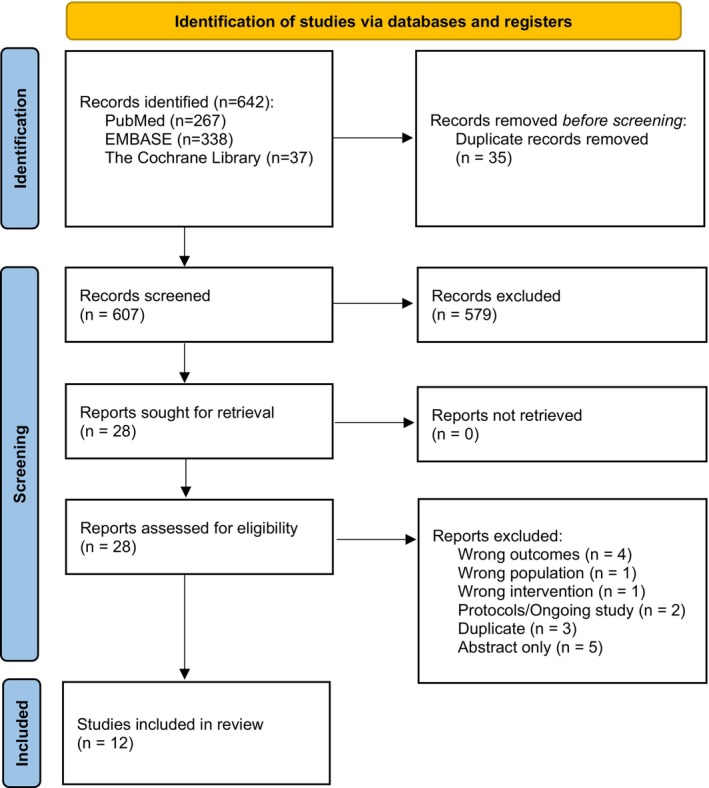
PRISMA flow diagram of included studies.

The study selection process is shown in Figure 1. After removal of duplicates, 607 records were screened, of which 28 reports were assessed in full text. Twelve studies met the eligibility criteria and were included in the final review. Detailed reasons for exclusion at the full‐text stage are provided in Table S2.

The characteristics of the included studies are presented in Tables 1, 2 and 3. In terms of the disease nomenclature used in the original reports, five studies (42%) explicitly adopted the recent MASLD criteria, while seven studies (58%) used the previous NAFLD definition. Overall, three studies were RCTs, and nine were NRSIs, including retrospective and prospective cohorts, as well as quasi‐experiments. With regard to the study protocols, three studies employed subcutaneous semaglutide, two utilised oral semaglutide, three employed subcutaneous liraglutide, one employed subcutaneous exenatide, one employed subcutaneous dulaglutide, and two studies evaluated more than one type of GLP‐1RA within their protocols. Despite including GLP‐1/GIP dual agonists in the search strategy, no eligible studies evaluating these agents were identified.

**TABLE 1:** Studies that evaluated the effects of GLP‐1 receptor agonists on muscle mass.

Study	Design	Population characteristics	Baseline demographics	*n*	Duration	Intervention	Weight change	Muscle unit of measure	Δ mean (±SD or 95% CI)	*p*‐value	Statistical direction
**Quantity assessment of muscle mass by CT/MRI**											
Ditzenberger, 2025 [33]	NRSI	MASLD; HIV; central adiposity	37% female; Age: 50.0 (11.0); BMI: 35.5 (5.6).	47	24w	Semaglutide, sc up to 1 mg/w	↓	Psoas muscle volume (mL)	−1.49 (−2.15, −0.83)	< 0.001	↓
Ishikawa, 2024 [23]	NRSI	NAFLD; T2D	67% female; Age: 67.3 (61.9, 74.5); BMI: 25.2 (21.8, 25.8).	15	48w	Semaglutide, po up to 7 mg/d	↓	SMI (cm^2^/m^2^)	NR	0.550	•
Kakegawa, 2024 [24]	NRSI	MASLD; T2D	48% female; Age: 52.0 (49.0, 64.0); BMI: 28.9 (26.3, 34.8).	21	24w	Semaglutide, sc up to 1 mg/w	•	SMI (cm^2^/m^2^)	NR	NS	•
Liu, 2023 [25]	RCT secondary analysis	NAFLD; T2D	46% female; Age: 47.6 (10.1); BMI: 28.5 (3.0).	35/36	24w	Exenatide, up to 10 μg 2×/d	NR	Psoas muscle area (mm^2^)	−149.09 (−322.90, 56.39)	0.214 (between‐group)	•
**Quantity assessment of muscle mass by DXA**											
Das, 2025 [26]	NRSI	MASLD; T2D; overweight/ obesity	66% female; Age: 50.8 (10.1); BMI: 34.3 (6.2).	36	52w	Semaglutide, po up to 14 mg/d	NR	ALM/Height^2^ (kg/m2)	0.7 (NR)	0.01	↑
Feng, 2019 [27]	RCT	NAFLD; T2D	28% female; Age: 46.8 (9.7); BMI: 28.1 (3.2).	29/29/27	24w	Liraglutide, sc up to 1.8 mg/d	↓	Total lean tissue (kg)	−0.2 (NR)	0.140 (between‐group)	•
Moolla, 2025 [28]	RCT	MASLD	47% female; Age: 48.0 (15.5); BMI: 35.7 (6.6).	15/14	12w	Liraglutide, sc up to 1.8 mg/d	↓	Total lean tissue (kg)	−1.4 (NR)	< 0.01	•
**Quantity assessment of muscle mass by BIA**											
Mittag‐ Roussou, 2020 [29]	NRSI	NAFLD; T2D	53% female; Age: 66.0 (56.0, 68.0); BMI: 34.8 (33.0, 38.7).	15/21	24w	Exenatide, sc, up to 10 μg 2×/d Liraglutide, sc up to 1.8 mg/d Dulaglutide, sc up to 1.5 mg/w	↓	Free fat mass (kg)	NR	0.296	•
Seko, 2017 [30]	NRSI	NAFLD; T2D	80% female; Age: 66.8 (2.7); BMI: 28.2 (1.2).	5	12w	Dulaglutide, sc 0.75 mg/w	↓	SMI (kg/m^2^)	−0.1 (NR)	0.500	•
So, 2025 [31]	NRSI	MASLD	50% female; Age: 46.3 (11.6); BMI: 30.9 (3.8).	192/192	24w	Liraglutide, sc dose NR	↓	ASM/Height^2^ (kg/m2)	−0.1 (0.0)	0.504 (between‐group)	•
Volpe, 2022 [32]	NRSI	NAFLD; T2D	46% female; Age: 57.7 (8.4); BMI: 38.8 (8.3).	46	48w	Semaglutide, sc up to 1 mg/w	↓	SMI (kg/m^2^)	−1.3 (2.0)	< 0.01	↓

*Note:* Baseline characteristics refer to the GLP‐1RA group and are expressed as mean (standard deviation) or median (interquartile range), according to the original study. Unless otherwise indicated, *p*‐values refer to within‐group changes (from baseline to end of treatment). (between‐group) indicates *p*‐values for the comparison between the intervention and control groups. Arrows (↑/↓) indicate the direction of statistically significant changes. A bullet (•) denotes non‐significant results, while numerical changes are reported as in the original study.

Abbreviations: ALM, appendicular lean mass; ASM, appendicular skeletal muscle; BIA, bioelectrical impedance analysis; BMI, body mass index (kg/m2); CT, computed tomography; d, day; DXA, dual‐energy X‐ray absorptiometry; HIV, human immunodeficiency virus; MASLD, metabolic dysfunction–associated steatotic liver disease; MRI, magnetic resonance imaging; NAFLD, nonalcoholic fatty liver disease; NR, not reported; NRSI, non‐randomised study of interventions; NS, non‐significant; po, per os; RCT, randomised controlled trial; sc, subcutaneous; SMI, skeletal muscle index; SMM, skeletal muscle mass; T2D, type 2 diabetes; w, week; Δ, delta; n, number of participants analysed (intervention/controls); ↑, increase; ↓, decrease.

**TABLE 2:** Studies that evaluated the effects of GLP‐1 receptor agonists on muscle strength.

Study	Design	Population characteristics	Baseline demographics	*n*	Duration	Intervention	Weight change	Muscle unit of measure	Δ mean (±SD or 95% CI)	*p*‐value	Statistical direction
**Strength assessment by Handgrip**											
So, 2025 [31]	NRSI	MASLD	50% female; Age: 46.3 (11.6); BMI: 30.9 (3.8).	192/192	24w	Liraglutide, sc dose NR	↓	kg	RH: −0.2 (NR) LH: −0.4 (NR)	NR	•
Volpe, 2022 [32]	NRSI	NAFLD; T2D	46% female; Age: 57.7 (8.4); BMI: 38.8 (8.3).	46	48w	Semaglutide, sc up to 1 mg/w	↓	kg	−0.3 (10.17)	NS	•
**Strength assessment by 5‐times sit‐to‐stand test**											
Ditzenberger, 2025 [33]	NRSI	MASLD; HIV; central adiposity	37% female; Age: 50.0 (11.0); BMI: 35.5 (5.6).	47	24w	Semaglutide, sc up to 1 mg/w	↓	sec	−0.66 (−1.4, 0.07)	0.077	•
**Strength assessment by 10‐times sit‐to‐stand test**											
Ditzenberger, 2025 [33]	NRSI	MASLD; HIV; central adiposity	37% female; Age: 50.0 (11.0); BMI: 35.5 (5.6).	47	24w	Semaglutide, sc up to 1 mg/w	↓	sec	−1.27 (−2.7, 0.10)	0.069	•

*Note:* Baseline characteristics refer to the GLP‐1RA group and are expressed as mean (standard deviation) or median (interquartile range), according to the original study. Arrows (↑/↓) indicate the direction of statistically significant changes. A bullet (•) denotes non‐significant results, while numerical changes are reported as in the original study. For time‐based outcomes, a negative delta reflects faster execution.

Abbreviations: BMI, body mass index (kg/m2); HIV, human immunodeficiency virus; kg, kilograms; LH, left hand; MASLD, metabolic dysfunction–associated steatotic liver disease; NAFLD, nonalcoholic fatty liver disease; NR, not reported; NRSI, non‐randomised study of interventions; NS, non‐significant; RH, right hand; sc, subcutaneous; sec, seconds; T2D, type 2 diabetes; w, week; n, number of participants analysed (intervention/control); Δ, delta; ↑, increase; ↓, decrease.

**TABLE 3:** Studies that evaluated the effects of GLP‐1 receptor agonists on muscle quality.

Study	Design	Population characteristics	Baseline demographics	*n*	Duration	Intervention	Weight change	Muscle unit of measure	Δ mean (±SD or 95% CI)	*p*‐value	Statistical direction
Quality assessment of skeletal muscle by MRI proton density fat fraction (MRI‐PDFF)											
Ditzenberger, 2025 [33]	NRSI	MASLD; HIV; central adiposity	37% female; Age: 50.0 (11.0); BMI: 35.5 (5.6).	47	24w	Semaglutide, sc up to 1 mg/w	↓	Muscle fat fraction (%)	−0.42 (−1.00, 0.17)	0.16	•
Kakegawa, 2024 [24]	NRSI	MASLD; T2D	48% female; Age: 52.0 (49.0, 64.0); BMI: 28.9 (26.3, 34.8).	21	24w	Semaglutide, sc up to 1 mg/w	•	Muscle fat fraction (%)	NR	0.0416	↓
Quality assessment of skeletal muscle by Proton Magnetic Resonance Spectroscopy (^1^H‐MRS)											
Cuthbertson, 2012 [34]	NRSI	NAFLD; T2D; obesity	52% female; Age: 50 (10); BMI: 38.4 (5.6).	25	24w	Exenatide, sc, up to 10 μg 2×/d Liraglutide, sc up to 1.2 mg/d	↓	Intramyocellular lipid content (CH_2_/creatine)	Soleus: −5 (−31, 46) Tibialis: 0 (−38, 38)	Soleus: 0.4937 Tibialis: 0.8671	•
Functional assessment of muscle quality (strength‐to‐mass ratio)											
Volpe, 2022 [32]	NRSI	NAFLD; T2D	46% female; Age: 57.7 (8.4); BMI: 38.8 (8.3).	46	48w	Semaglutide, sc up to 1 mg/w	↓	HGS/SMM (kg/kg)	0.1 (0.1)	NS	•

*Note:* Baseline characteristics are expressed as mean (standard deviation) or median (interquartile range), according to the original study. Arrows (↑/↓) indicate the direction of statistically significant changes. A bullet (•) denotes non‐significant results, while numerical changes are reported as in the original study.

Abbreviations: BMI, body mass index (kg/m2); d, day; HGS, handgrip strength; HIV, human immunodeficiency virus; MASLD, metabolic dysfunction–associated steatotic liver disease; MRI, magnetic resonance imaging; NAFLD, nonalcoholic fatty liver disease; NR, not reported; NRSI, non‐randomised study of interventions; NS, non‐significant; sc, subcutaneous; SMM, skeletal muscle mass; T2D, type 2 diabetes; w, week; n, number of participants analysed (intervention/control); Δ, delta; ↓, decrease.

Of the 12 included studies, outcome assessment varied: Seven evaluated muscle mass alone, one evaluated muscle quality alone, one assessed both muscle mass and strength, one assessed muscle mass and quality, and two studies examined all three domains. The duration of the studies ranged from 12 to 52 weeks, with the majority lasting 24 weeks.

### 3.2 Risk of Bias Assessment

RoB 2 was applied to RCTs, most of which were rated as having some concerns (66.7%), mainly related to the randomisation process. For NRSIs, ROBINS‐I indicated an overall high risk of bias (above 75%), predominantly driven by serious confounding and, to a lesser extent, by selection of participants. Detailed domain‐level assessments are provided in Figures S1–S4.

### 3.3 Synthesis of Results

#### 3.3.1 Muscle Mass

Muscle mass was evaluated in 11 of the included studies, involving a total of 785 participants (466 in GLP‐1RAs groups and 319 in control groups). Results are presented in Table 1, according to the assessment tool used: CT/MRI, DXA, and BIA. Across studies, the predominant pattern was a numerical reduction in muscle mass, observed in nine cohorts, although the magnitude of change was generally small and statistically significant in only three of them.

In studies using CT or MRI, regarded as reference techniques for assessing skeletal muscle quantity/mass [14], GLP‐1RA therapy was generally associated with small changes in muscle mass. Three out of four studies reported no significant differences in skeletal muscle quantity, assessed either by Skeletal Muscle Index (SMI, cm^2^/m^2^)—calculated as cross‐sectional muscle area normalised to height squared—or by absolute muscle area (mm^2^). The only study reporting a statistically significant reduction in muscle mass showed a modest decrease in psoas muscle volume over 24 weeks. However, this finding was derived from a non‐randomised design without a comparator group and involved a population with MASLD and HIV.

DXA‐based assessments yielded heterogeneous results across three studies, reporting increases, decreases, or no detectable changes in lean mass outcomes. The proxies used within this method varied, including appendicular lean mass adjusted for height (ALM/height^2^, kg/m^2^) and total lean tissue (kg). Notably, the study reporting an increase in ALM/height^2^ showed a small absolute gain over a longer follow‐up period. For this specific study, no physical exercise co‐intervention was explicitly described in its protocol, although patients concurrently received calcium and vitamin D supplementation [26]. In contrast, Moolla et al. [28]. observed a reduction in lean mass that was both statistically significant and larger in magnitude than in the other DXA studies. Based on reported group mean values, the estimated proportion of total weight loss attributable to lean mass was approximately 4% in Feng et al. [27] (0.2 kg lean mass out of 5.6 kg body weight) and 27% in Moolla et al. [28] (1.4 kg lean mass out of 5.2 kg body weight). These estimates were derived from aggregate mean values and were not reported as predefined outcomes in the original studies.

Among the four studies evaluating muscle mass via BIA, three reported no significant reductions following GLP‐1RA therapy, including the study with the largest sample size (384 participants). The assessment proxies were heterogeneous, most commonly employing a skeletal muscle index (SMI, kg/m^2^), while one study used fat‐free mass (FFM, kg), an estimate generally considered less specific for skeletal muscle mass. Volpe et al. [32] reported a significant decrease in SMI after 48 weeks of semaglutide (up to 1 mg/week); however, in that study, muscle loss exceeding 40% of total weight reduction was reported only for a minority (18%) of participants.

#### 3.3.2 Muscle Strength

Muscle strength was assessed in two studies using HGS (*n* = 430; 238 in GLP‐1RAs groups and 192 in control groups) and in one study using both the 5‐times and 10‐times STS tests (*n* = 47; all in GLP‐1RAs groups) (Table 2). Based on reported estimates, muscle strength was largely preserved during GLP‐1RA therapy, despite clinically meaningful weight loss. Small numerical reductions in HGS were reported, but the magnitude of change was minimal. Notably, So et al. [31] did not report statistical testing for strength outcomes; however, the absolute numerical variation on HGS was marginal (−0.2 to −0.4 kg), consistent with preserved strength. In contrast, STS performance showed modest numerical improvements, indicating preserved functional capacity during weight loss.

#### 3.3.3 Muscle Quality

Muscle quality was assessed in four studies (*n* = 139; all in GLP‐1RAs groups), using imaging or functional metrics (Table 3). Among the three studies employing imaging tools, a consistent trend toward reduced muscle fat content was observed across different compartments. Specifically, two studies using MRI‐PDFF reported numerical reductions in muscle fat fraction, with one reaching statistical significance. The study utilising ^1^H‐MRS focused exclusively on intramyocellular lipids, also showing a non‐significant decrease, while extramyocellular lipid content was not reported. Complementing these imaging findings, Volpe et al. [32] assessed muscle quality through a functional lens, showing that the strength‐to‐mass ratio remained stable and suggesting that the force‐generating capacity per unit of muscle tissue was maintained. Collectively, these results indicate that GLP‐1RA‐induced weight loss does not adversely affect, and may potentially improve, skeletal muscle quality.

## 4 Discussion

This systematic review provides a comprehensive synthesis of current evidence on how GLP‐1RAs influence muscle outcomes in adults with MASLD. A substantial degree of heterogeneity was observed across studies, particularly in the methods used to quantify muscle mass. While most cohorts showed a downward trend in lean mass, these changes were generally small and statistically non‐significant, likely reflecting modest absolute losses rather than clinically relevant wasting. Importantly, muscle strength remained largely preserved across all functional assessments, and muscle quality showed a directionally consistent numerical improvement through reduced intramuscular fat infiltration, as well as preserved strength‐to‐mass ratio. Taken together, the available data suggest that GLP‐1RA therapy does not lead to clinically meaningful deterioration of muscle mass or function in adults with MASLD, although confirmation from higher‐quality studies using standardised assessment methods is warranted.

The observed relative preservation of muscle outcomes is particularly relevant given the susceptibility of individuals with MASLD to muscle impairment. Findings from the broader literature on incretin‐based therapies in obesity and type 2 diabetes show that quantitative reductions in lean mass typically account for 15% to 45% of total weight loss [35]. Landmark trials such as STEP‐1 (semaglutide) [36], SUSTAIN‐8 (semaglutide) [37], and SURMOUNT‐1 (tirzepatide) [38] offer contextual support for this estimate. Consequently, body composition can be preserved or improved even if there is some absolute loss of lean mass. Nevertheless, most comparative evidence is drawn from broader metabolic cohorts, potentially encompassing individuals with MASLD, yet lacking disease‐specific analyses. Importantly, no direct catabolic effect of GLP‐1RAs on skeletal muscle has been demonstrated; conversely, animal models suggest potential protective effects on muscle tissue within metabolic disease contexts [17, 39]. Furthermore, decreases in lean tissue observed during GLP‐1RA therapy are not unique to this drug class, as they are a well‐recognised component of weight loss achieved through dietary or surgical interventions [40].

According to the data from this systematic review, although numerical reductions in muscle mass were common, evidence for a clinically relevant loss remains limited. Based on MRI data, Ditzenberger et al. [33] observed a reduction in psoas volume; however, this finding derives from a population of individuals with HIV and MASLD undergoing antiretroviral therapy, which likely reflects a distinct clinical and metabolic‐inflammatory profile rather than a generalised effect of GLP‐1RA therapy. Conversely, among studies evaluating body composition by DXA or BIA, even in those reporting larger reductions, lean mass loss represented a smaller proportion of total weight loss. Volpe et al. [32] reported that a reduction in skeletal muscle index exceeding 40% of total weight loss occurred in only 18% of participants. Similarly, based on group mean estimates, lean mass accounted for 27% of total weight loss in Moolla et al. [28], which is within the range typically observed in other weight‐loss interventions [40]. These observations should be interpreted cautiously, given the small sample sizes, heterogeneous assessment methods, and limited follow‐up durations, but they indicate that lean mass reductions, when present, do not necessarily imply disproportionate or clinically relevant muscle wasting.

Beyond quantitative changes in muscle mass, functional and qualitative muscle outcomes may be more clinically meaningful in patients with MASLD. Muscle strength, in particular, is a stronger predictor of disability and adverse outcomes than muscle mass alone [6, 14, 41]. Although limited, the available evidence in this review does not indicate that GLP‐1RA therapy leads to impairment in muscle strength, as assessed by handgrip strength or sit‐to‐stand performance. These findings align with the broader GLP‐1RA literature, in which this drug class has generally been considered neutral or even beneficial with respect to muscle strength [17, 42]. Nevertheless, data from high‐risk populations—such as older adults with diabetes and a high prevalence of sarcopenia—have raised concerns about potential strength decline in specific contexts [43]. Given the scarcity of MASLD‐specific data, conclusions regarding muscle strength should therefore be interpreted cautiously.

Another consideration is whether the apparent preservation of muscle strength reflects a pre‐existing low functional reserve in patients with MASLD, potentially creating a floor effect. However, available data suggest this is unlikely. Baseline strength values in the included studies—such as mean HGS ranging from 30.8 to 33.6 kg [31, 32] and a mean 5‐times STS time of 12.5 s [33]—generally exceed established clinical cutoffs for sarcopenia (e.g., HGS < 27 kg for men and < 16 kg for women, or STS > 15 s) [14]. Although the prevalence of sarcopenia was not reported in the primary studies, these values indicate that participants retained adequate functional reserve. Thus, the stability of muscle strength during GLP‐1RA therapy is unlikely to be explained by depleted baseline muscle capacity.

Finally, muscle quality—most commonly reflected by myosteatosis—represents another clinically relevant dimension in MASLD [44], even in the absence of obesity [45]. Persistent adipose inflammation promotes lipid redistribution toward visceral and skeletal muscle depots, increasing free fatty acid flux and contributing to both MASLD progression and intramuscular fat accumulation [46]. Although MASLD‐specific data on myosteatosis remain limited, findings from populations with obesity and/or type 2 diabetes have shown that incretin‐based therapies tend to reduce intramuscular fat [47, 48]. These observations provide contextual support, even though they do not replace the MASLD‐specific evidence synthesised in this review.

Among the included studies, there was a tendency toward improved muscle quality via reduced myosteatosis. Although these findings require confirmation in larger and more rigorously designed studies, a reduction in muscle fat infiltration would be consistent with the broader pattern observed in this review: Weight loss induced by GLP‐1RAs is associated with modest reductions in lean mass, but appears to spare muscle function and could potentially enhance muscle quality through improvements in intramuscular lipid content, as suggested by Kakegawa et al. [24]. A complementary perspective comes from functional assessments of muscle quality, which also showed reassuring results. This is further supported by data from bariatric surgery, which often shows preservation of grip strength despite significant reductions in lean mass [49], reinforcing that decreases in lean mass do not necessarily translate into functional impairment. Ultimately, reductions in lean mass, particularly when estimated by DXA or BIA, should not be interpreted as loss of contractile tissue alone; these methods cannot distinguish muscle from non‐contractile components [50], so shifts in hydration status or reductions in intramuscular fat may be misclassified as lean‐mass loss. In this context, part of the observed decline could reflect improvements in muscle composition rather than true loss of muscle tissue.

The scope and generalisability of our findings are constrained by several limitations. First, the number of available studies was limited, and most included small sample sizes, non‐randomised designs, and a high risk of bias, substantially limiting statistical power and the ability to detect small‐to‐moderate effects on muscle‐related outcomes. Second, GLP‐1RA interventions varied substantially in drug type, dosing, and treatment duration. Crucially, nearly all studies used glycemic‐control doses and short treatment periods, rather than the higher, obesity‐approved regimens now standard in clinical practice, which are typically used for much longer durations in real‐world settings. This mismatch substantially limits the generalisability of the findings and may partly explain the absence of significant weight reduction in some studies, thereby potentially influencing muscle‐related outcomes. Third, many studies also lacked control for important confounders of the outcome, such as diet and physical activity. In addition, population characteristics differed markedly across the included cohorts, with substantial variation in age, sex distribution, BMI, and diabetes status. These factors are known to influence muscle phenotype and likely contributed to the observed heterogeneity in outcomes. Finally, the assessment of muscle outcomes lacked methodological standardisation across studies, with substantial variability in imaging modalities, surrogate measures, and reporting units. In particular, several studies relying on DXA or BIA used total lean mass or fat‐free mass as proxies for skeletal muscle, which may overestimate true muscle tissue by including non‐muscle, non‐fat components.

Despite these limitations, this systematic review has notable strengths. To our knowledge, it represents the first comprehensive synthesis specifically addressing the effects of GLP‐1RA therapy on skeletal muscle mass, strength, and quality in adults with MASLD. The inclusion of multiple muscle‐related domains and the stratification of results according to assessment modality provide a nuanced interpretation of the existing literature.

From a clinical standpoint, while definitive conclusions cannot yet be drawn, current evidence does not suggest clinically relevant muscle wasting in adults with MASLD receiving GLP‐1RAs. Nonetheless, given the intrinsic vulnerability of this population to muscle impairment, clinicians should consider individualised patient monitoring, particularly for high‐risk groups such as those with advanced liver disease, older age, or pre‐existing sarcopenia. Furthermore, lifestyle strategies such as resistance exercise combined with adequate protein intake are essential for preserving lean mass and functional capacity during pharmacologically induced weight loss [35]. Additionally, evidence suggests that combining supervised exercise with pharmacotherapy may promote healthier long‐term weight maintenance, with benefits persisting even after treatment discontinuation, as observed in populations with obesity [51].

Beyond synthesising current evidence, this review highlights significant gaps in the available literature. Notably, very few studies have performed longitudinal assessments of muscle strength, and none have evaluated muscle mass, quality, and function in a paired, site‐specific manner within the same muscle groups. This methodological limitation precludes a definitive assessment of the relationship between structural, compositional, and functional muscle changes. Integrated approaches that combine imaging‐based assessments of muscle mass and quality with functional measurements of the corresponding muscle groups (e.g., isokinetic dynamometry for knee extension alongside quadriceps cross‐sectional area and composition) would help generate more comparable and clinically meaningful evidence.

In conclusion, the available evidence does not indicate a consistent or disproportionate negative effect of GLP‐1RA therapy on muscle mass, strength, or quality in adults with MASLD. Although modest reductions in lean mass may occur during weight loss, these changes are generally proportional to overall weight reduction and are not accompanied by functional decline. Furthermore, early data suggest potential improvements in muscle quality through reductions in intramuscular fat. Despite these reassuring findings, the evidence base remains limited, and high‐quality trials in patients with MASLD, using standardised muscle assessments, are needed to clarify the long‐term effects of GLP‐1RAs on skeletal muscle health.

## Author Contributions

F.I. and P.M. were responsible for the study conception and design. F.I. and T.J. contributed to data curation, screening, and extraction. F.I. drafted the original manuscript. All authors contributed to data interpretation. M.Y., M.R.T., B.D.S., and P.M. critically evaluated the manuscript for important intellectual content. All authors revised and approved the final version of the manuscript.

## Funding

This work was supported by the Coordenação de Aperfeiçoamento de Pessoal de Nível Superior, Finance Code 001.

## Conflicts of Interest

The authors declare no conflicts of interest.

## Supporting information


**Figure S1:** Summary Plot of Risk of Bias for Randomised Control Trials (Rob2 Tool).
**Figure S2:** Traffic Light Plot of Risk of Bias for Randomised Control Trials (Rob2 Tool).
**Figure S3:** Summary Plot of Risk of Bias for Non‐Randomised Studies of Intervention (Robins‐I Tool).
**Figure S4:** Traffic Light Plot of Risk of Bias for Non‐Randomised Studies of Intervention (Robins‐I Tool).
**Table S1:** Search strategy.
**Table S2:** List of excluded reports with exclusion reasons.

## Data Availability

The data that support the findings of this study are available from the corresponding author upon reasonable request.
