# Supplementary material for: Effects of GLP‐1 Receptor Agonists on Muscle Mass, Strength, and Quality in MASLD: A Systematic Review
Source: Liver Int. 2026 Apr 17;46(5):e70643. doi: 10.1111/liv.70643 (PMC13090617; doi:10.1111/liv.70643)

**SUPPLEMENTARY MATERIAL**

**Article:** Effects of GLP‑1 Receptor Agonists on Muscle Mass, Strength, and Quality in MASLD: A Systematic Review

**Authors names:** Fernando Iorra*, Tanya Jayakar, Michael Yee, Mark R. Thursz, Beatriz D. Schaan, Pinelopi Manousou*

** Corresponding authors: endocrino.fernando@gmail.com; p.manousou@imperial.ac.uk*

**Table S1. Search strategy**

| Pubmed  (n = 267) | (Fatty Liver[mh:noexp] OR Non-alcoholic Fatty Liver Disease[mh] OR Non alcoholic Fatty Liver*[tiab] OR Nonalcoholic Fatty Liver*[tiab] OR Nonalcoholic Steatohepa*[tiab] OR NAFLD[tiab] OR NASH[tiab] OR MAFLD[tiab] OR MASLD[tiab] OR MASH[tiab] OR metabolic dysfunction associated steatohepatitis[tiab] OR "Metabolic Liver Disease"[tiab:~5] OR "Metabolic Liver Diseases"[tiab:~5])  AND  (Glucagon-Like Peptide 1[mh] OR Glucagon Like Peptide 1[tiab] OR GLP 1*[tiab] OR GLP1*[tiab] OR Glucagon-Like Peptide-1 Receptor Agonists[mh] OR Incretin Mimetics[tiab] OR Glucagon-Like Peptide-1 Receptor[mh] OR Glucagon-Like Peptide-1 Receptor Agonists[pa] OR "rGLP-1 protein"[tiab] OR dulaglutide[tiab] OR semaglutide[tiab] OR Liraglutide[tiab] OR Exenatide[tiab] OR Tirzepatide[tiab] OR Victoza[tiab] OR Saxenda[tiab] OR "NN 2211"[tiab] OR "NN2211"[tiab] OR Trulicity[tiab] OR Byetta[tiab] OR Rybelsus[tiab] OR Wegovy[tiab] OR Ozempic[tiab] OR Mounjaro[tiab] OR “LY3298176”[tiab] OR Zepbound[tiab])  AND  (Muscle Strength[mh] OR Muscles[mh] OR Musc*[tiab] OR Streng*[tiab] OR Body Constitution[mh] OR Body Constitution*[tiab] OR Body Composition*[tiab] OR Body Mass[tiab] OR Sarcopenia[mh] OR Sarcopenia*[tiab]) |
| --- | --- |
| EMBASE  (n = 338) | ('fatty liver'/de OR 'nonalcoholic fatty liver'/exp OR ('non alcoholic fatty liver*' OR 'nonalcoholic fatty liver*' OR 'nonalcoholic steatohepa*' OR nafld OR nash OR mafld OR masld OR mash OR 'metabolic dysfunction associated steatohepatitis' OR (metabolic NEAR/6 liver NEAR/6 disease) OR (metabolic NEAR/6 liver NEAR/6 diseases)):ti,ab,kw)  AND  (('glucagon like peptide 1' OR 'glucagon like peptide 1 receptor agonist' OR 'glucagon like peptide 1 receptor')/exp OR ('glucagon like peptide 1' OR 'glp 1*' OR 'glp1*' OR 'incretin mimetics' OR 'rglp-1 protein' OR dulaglutide OR semaglutide OR liraglutide OR exenatide OR tirzepatide OR victoza OR saxenda OR 'nn 2211' OR 'nn2211' OR trulicity OR byetta OR rybelsus OR wegovy OR ozempic OR mounjaro OR 'ly3298176' OR zepbound):ti,ab,kw)  AND  (('muscle strength' OR muscle OR 'body constitution' OR sarcopenia)/exp OR (musc* OR streng* OR 'body constitution*' OR 'body composition*' OR 'body mass' OR sarcopenia*):ti,ab,kw)  AND [embase]/lim NOT ([embase]/lim AND [medline]/lim) |
| The Cochrane Library  (n = 37) | ([mh ^"Fatty Liver"] OR [mh "Non alcoholic Fatty Liver Disease"] OR (("Non alcoholic Fatty" NEXT Liver*) OR ("Nonalcoholic Fatty" NEXT Liver*) OR (Nonalcoholic NEXT Steatohepa*) OR NAFLD OR NASH OR MAFLD OR MASLD OR MASH OR "metabolic dysfunction associated steatohepatitis" OR (Metabolic NEAR/5 Liver NEAR/5 Disease) OR (Metabolic NEAR/5 Liver NEAR/5 Diseases)):ti,ab,kw)  AND  ([mh "Glucagon Like Peptide 1"] OR [mh "Glucagon Like Peptide-1 Receptor Agonists"] OR [mh "Glucagon Like Peptide-1 Receptor"] OR ("Glucagon Like Peptide 1" OR (GLP NEXT 1*) OR GLP1* OR "Incretin Mimetics" OR "rGLP-1 protein" OR dulaglutide OR semaglutide OR Liraglutide OR Exenatide OR Tirzepatide OR Victoza OR Saxenda OR "NN 2211" OR "NN2211" OR Trulicity OR Byetta OR Rybelsus OR Wegovy OR Ozempic OR Mounjaro OR "LY3298176" OR Zepbound):ti,ab,kw)  AND  ([mh "Muscle Strength"] OR [mh Muscles] OR [mh "Body Constitution"] OR [mh Sarcopenia] OR (Musc* OR Streng* OR (Body NEXT Constitution*) OR (Body NEXT Composition*) OR "Body Mass" OR Sarcopenia*):ti,ab,kw)  NOT ((Embase):an OR (Pubmed):an) |

**Table S2. List of excluded reports with exclusion reasons**

| **First author, year** | **Title** | **Exclusion reason** |
| --- | --- | --- |
| Akhverdyan, 2024 | Changes in Transient Elastography with Glucagon-Like Peptide-1 Receptor Agonist Use in Metabolic Dysfunction Associated Steatotic Liver Disease: A Real-World Retrospective Analysis | Wrong outcome |
| Armstrong, 2010 | Effects of two years of liraglutide treatment on fatty liver disease in patients with type 2 diabetes: Analysis of the lead-2 extension trial | Abstract only |
| Armstrong, 2011 | Effects of once-daily liraglutide on fatty liver disease in patients with type 2 diabetes (T2D) after 2 years' treatment: Retrospective-analysis of the lead-2 extension trial | Abstract only |
| Armstrong, 2016 | Liraglutide safety and efficacy in patients with non-alcoholic steatohepatitis (LEAN): a multicentre, double-blind, randomised, placebo-controlled phase 2 study | Wrong outcome |
| Stratina, 2024 | NewInsights on Using Oral Semaglutide versus Dapagliflozin in Patients with Type 2 Diabetes and Metabolic Dysfunction-Associated Steatotic Liver Disease | Wrong outcome |
| Cuthbertson, 2012 | Glucagon-like receptor 1 (GLP-1) analogues reduce liver fat content in patients with Type 2 diabetes: A LIVERpool magnetic resonance spectroscopy study | Duplicate |
| Das, 2024 | Effect of Oral Semaglutide on Hepatic Steatosis and Fibrosis, and Bone Microarchitecture in Non-alcoholic Fatty Liver Disease Complicating Type 2 Diabetes Mellitus: A Prospective, Interventional, Open-Label, Paired Study | Duplicate |
| Farrell, 2024 | Effects of the very low energy diet compared to a mediterranean diet on hepatic and metabolic outcomes in metabolic associated steatotic liver disease: a randomised control trial | Wrong intervention |
| Feng, 2018 | Effect of liraglutide, metformin, and gliclazide on body composition in type 2 diabetic patients with nonalcoholic fatty liver disease-a randomised trial | Duplicate |
| Foghsgaard, 2023 | Liraglutide treatment for the prevention of glucose tolerance deterioration in women with prior gestational diabetes mellitus: A 52-week randomized controlled clinical trial | Wrong patient population |
| Fogla, 2024 | Semaglutide use achieves weight loss and fat reduction with preservation of skeletal mass in patients with MASLD: a retrospective longitudinal study | Abstract only |
| Miyake, 2022 | Additional Effect of Luseogliflozin on Semaglutide in Nonalcoholic Steatohepatitis Complicated by Type 2 Diabetes Mellitus: An Open-Label, Randomized, Parallel-Group Study | Protocol/Ongoing study |
| Parzer, 2025 | The effects of a two-week hypocaloric meal replacement compared to an established liraglutide therapy on body composition and non-invasive markers of MAFLD before bariatric surgery | Abstract only |
| Wong, 2022 | Semaglutide used in adjunct to multidisciplinary lifestyle interventions leads to weight reduction and improvement in body composition in patients with NAFLD | Abstract only |
| Yan, 2019 | Liraglutide, Sitagliptin, and Insulin Glargine Added to Metformin: The Effect on Body Weight and Intrahepatic Lipid in Patients With Type 2 Diabetes Mellitus and Nonalcoholic Fatty Liver Disease | Wrong outcome |
| EUCTR2018‐002162‐38‐FR | A study comparing the effect of Dulaglutide (TRULICITY®) add-on to dietary reinforcement versus dietary reinforcement alone in patients with type 2 diabetes and carriers of a non-alcoholic steatohepatitis | Protocol/Ongoing study |

**BIAS ASSESSMENT**

**Figure S1. Summary Plot of Risk of Bias for Randomized Control Trials (Rob2 Tool)**


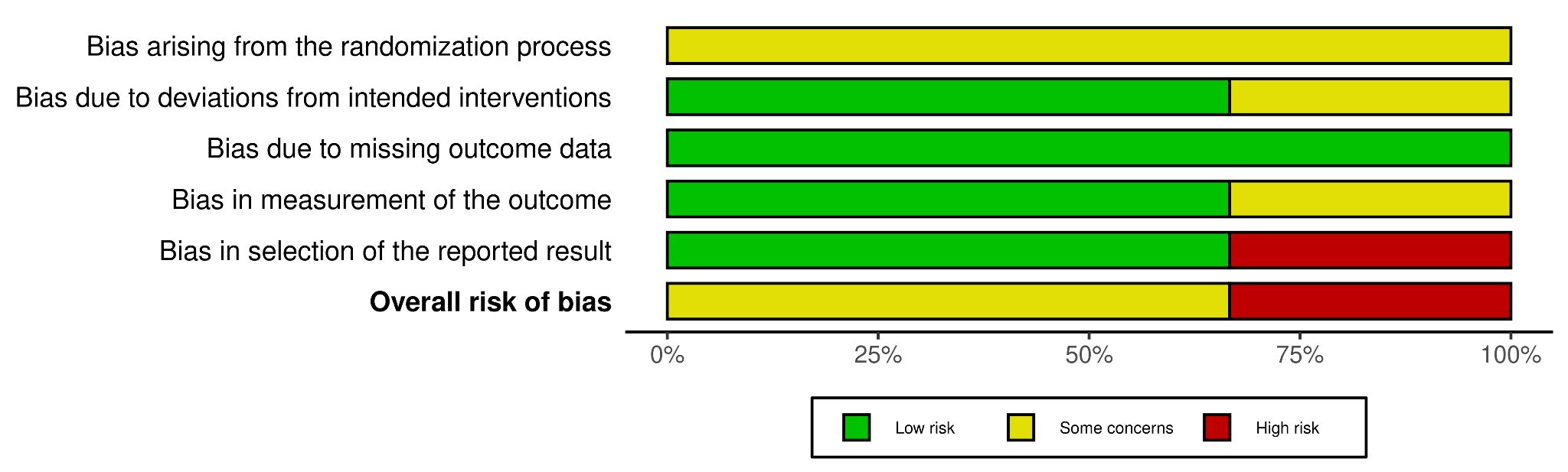


**Figure S2. Traffic Light Plot of Risk of Bias for Randomized Control Trials (Rob2 Tool)**

**
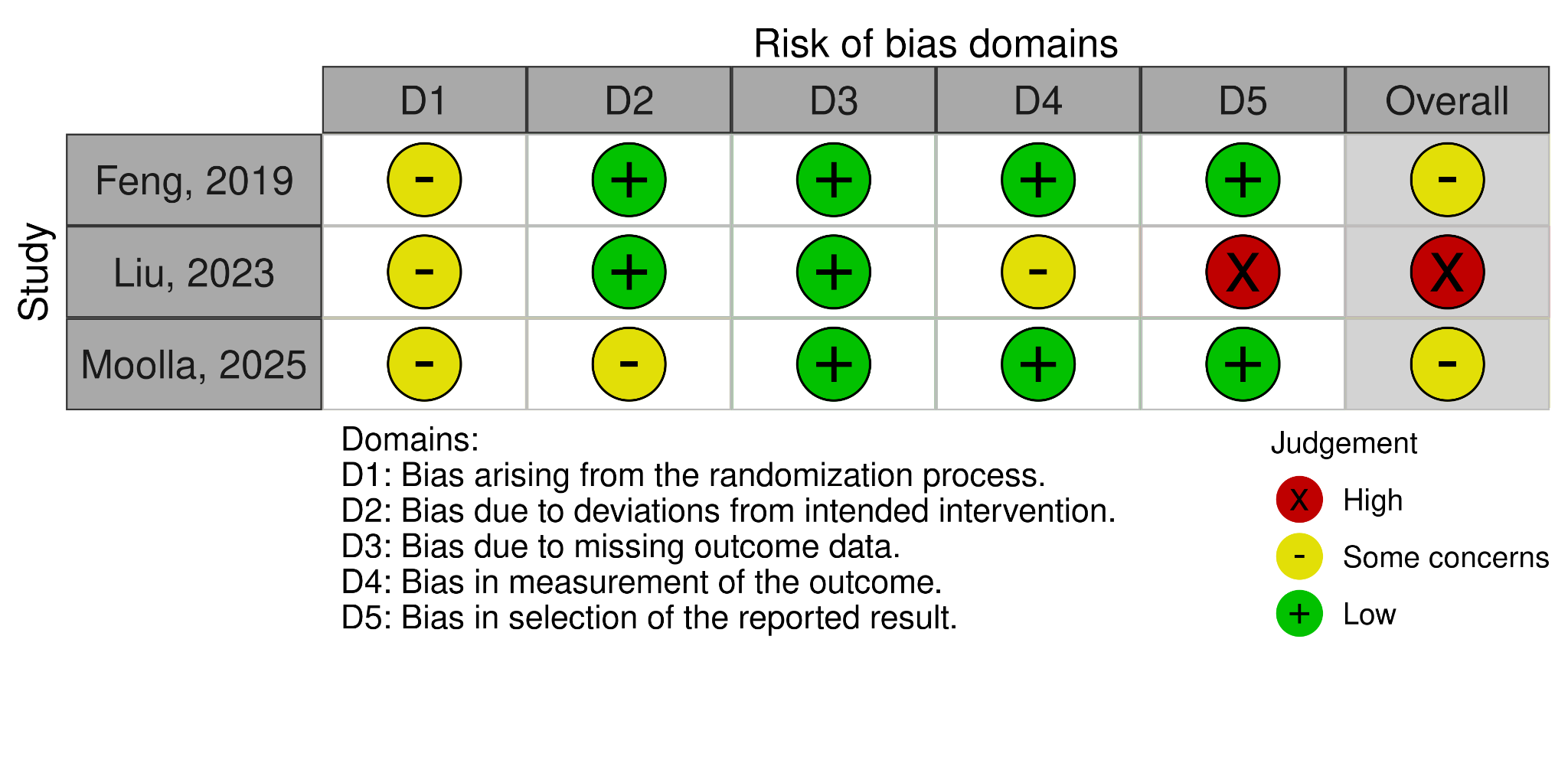
**

**Figure S3. Summary Plot of Risk of Bias for Non-Randomized Studies of Intervention (Robins-I Tool)**

**
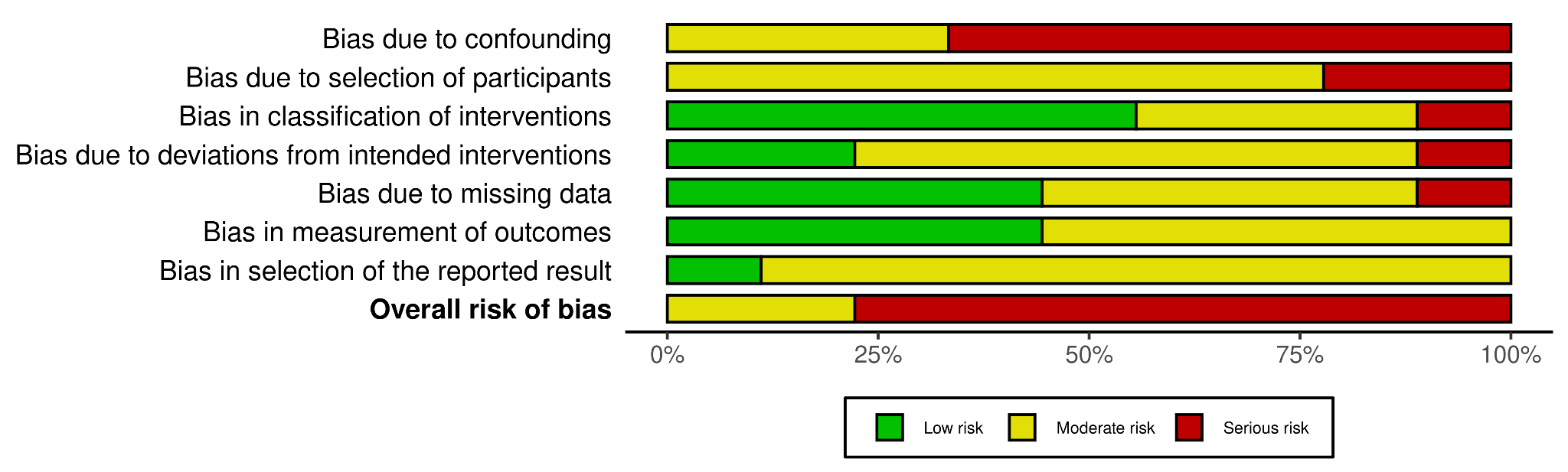
**

**Figure S4. Traffic Light Plot of Risk of Bias for Non-Randomized Studies of Intervention (Robins-I Tool)**


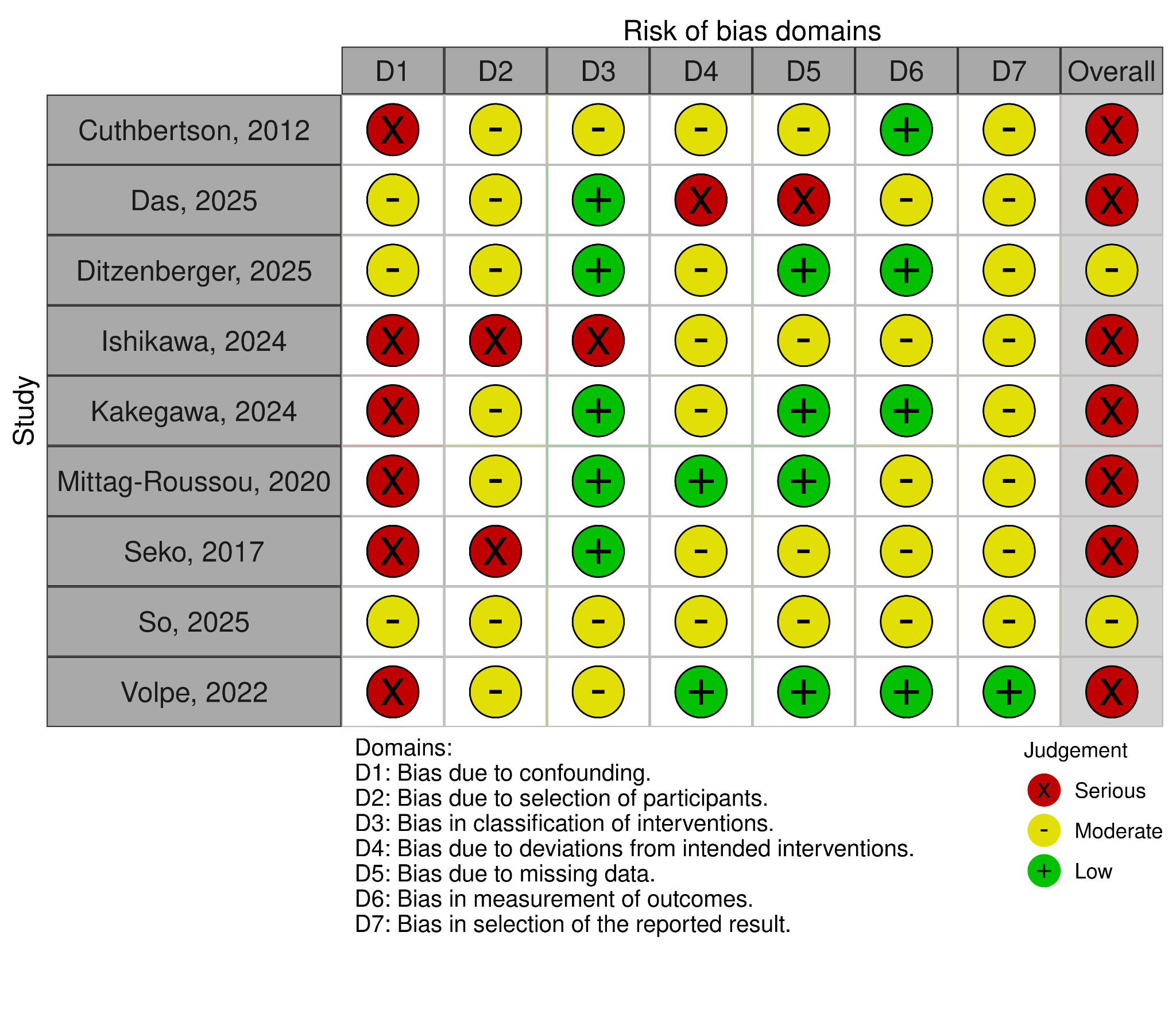

Supplement: Supplementary file 1 — Figure S1: Summary Plot of Risk of Bias for Randomised Control Trials (Rob2 Tool). Figure S2: Traffic Light Plot of Risk of Bias for Randomised Control Trials (Rob2 Tool). Figure S3: Summary Plot of Risk of Bias for Non‐Randomised Studies of Intervention (Robins‐I Tool). Figure S4: Traffic Light Plot of Risk of Bias for Non‐Randomised Studies of Intervention (Robins‐I Tool). Table S1: Search strategy. Table S2: List of excluded reports with exclusion reasons. [file LIV-46-0-s001.docx]
